# ARETTA: Assessing Response to Neoadjuvant Taxotere and Subcutaneous Trastuzumab in Nigerian Women With HER2-Positive Breast Cancer: A Study Protocol

**DOI:** 10.1200/GO.20.00043

**Published:** 2020-07-06

**Authors:** Atara I. Ntekim, Abiola Ibraheem, Adenike A. Sofoluwe, Olayinka Kotila, Chinedum Babalola, Theodore Karrison, Christopher O. Olopade

**Affiliations:** ^1^ Department of Radiation Oncology, College of Medicine, University of Ibadan, Ibadan, Nigeria; ^2^ Section of Hematology Oncology, University of Chicago, Chicago, IL; ^3^ Department of Radiology, College of Medicine, University of Ibadan, Ibadan, Nigeria; ^4^ Department of Pharmaceutical Chemistry, Faculty of Pharmacy, University of Ibadan, Ibadan, Nigeria; ^5^ Department of Public Health Sciences, University of Chicago, Chicago, IL; ^6^ Center for Global Health, University of Chicago, Chicago, IL

## Abstract

Human epidermal growth factor receptor 2 (HER2) subtype of breast cancer is aggressive, leading to a poor outcome. Targeted therapy with trastuzumab has been shown to be effective in the treatment of HER2-positive breast cancer. Cardiotoxicity is a specific adverse effect associated with trastuzumab. The initial formulation of trastuzumab was intravenous, but presently, a subcutaneous formulation (Herceptin SC) is available. Insufficient data on the response rate and cardiotoxic effects of trastuzumab among indigenous Black populations exist. In all studies evaluating the efficacy and toxicity of trastuzumab alone or in combination with chemotherapy, indigenous Black populations in Africa were not included, yet they are the ones most likely to benefit from highly effective cancer medicines. This is partly due to poor oncology clinical trial infrastructure in sub-Saharan Africa. The ARETTA study protocol (ClinicalTrials.gov identifier: NCT03879577) is a phase II multicenter feasibility study to evaluate the efficacy and toxicity of docetaxel given every 3 weeks for 4 cycles plus trastuzumab in 60 previously untreated women with nonmetastatic breast cancer. The primary endpoint is to assess the proportion of patients with complete pathologic response. Secondary endpoints include the number of patients who require dose delays in docetaxel and trastuzumab attributed to hematologic, GI, and cardiac toxicity. Pharmacokinetic profiles of subcutaneous trastuzumab will also be determined. The ARETTA study will provide important information on the clinical response and cardiac safety of subcutaneous trastuzumab in combination with docetaxel among indigenous African women with nonmetastatic breast cancer. It can also be used as a blueprint for conducting biomarker-driven oncology clinical trials in low-resource settings such as sub-Saharan Africa.

## INTRODUCTION

The incidence of cancer in sub-Saharan Africa (SSA) is increasing, with high mortality. Racial/ethnic disparities in cancer mortality continue to widen, and biomarker-intensive clinical trials rarely investigate patients with cancer in underserved populations of African ancestry. Few studies that describe differences in genomic characteristics of malignancies among people of African descent compared with Whites exist, implying the need for an individualized treatment approach.^1^

**CONTEXT**

**Key Objective**
Can more oncology clinical trials be conducted in sub-Saharan Africa? This study is an example of a simple, feasible, and suitable protocol that can be implemented in sub-Saharan Africa.
**Knowledge Generated**
The study protocol will determine the response and toxicity profile of docetaxel and subcutaneous Herceptin (trastuzumab) in Nigerian women with human epidermal growth factor receptor 2 (HER2)-positive breast cancer. The outcome of the study will provide local data that will guide oncologists in choice of chemotherapy for patients with HER2-positive breast cancer in Nigeria and sub-Saharan Africa.
**Relevance**
The implementation of this protocol will enable investigators in sub-Saharan Africa to build clinical trial teams and gain experience in oncology clinical trials.

Clinical trials through which evidence for effective therapies are obtained are scarce in SSA. Moreover, most cancer drugs currently in use in SSA were never tried in the region before approval. Among the study population, there is a scarcity of data on the response rate, toxicity, and quality of life (QoL) during treatment with any cancer therapy currently in use. Ethnic and population variations exist with respect to drug response and toxicity.^2^ There is a need to bring oncology clinical trials into SSA to provide evidence for treatment choice. Without optimal chemotherapy, cancer mortality, including breast cancer mortality in the Nigerian population, will remain high. In 2018, the estimated number of newly diagnosed patients with breast cancer in Nigeria was 26,310, constituting approximately 23% of all patients with cancer. Of these, 11,564 (45%) of patients with breast cancer died as a result of the disease.^3^ Ensuring the development of cancer research that meets the needs of people who live in low-resource settings is integral to alleviating the cancer burden. There is a view that a focus on phase II and III cancer clinical trials in SSA can give African researchers the opportunity to “learn by doing” to foster sustainable clinical research capacity through international collaboration between African partners and developed countries, and regional collaboration among African research institutes.^4^ Research collaborations between African and non-African partners hold the potential to have a significant impact on the development of clinical research capacity in SSA. This study is a collaboration between the University of Chicago and 4 centers in Nigeria.

The lack of participation of Black patients in clinical trials prevents the application of clinical trial results to the global population.^5^ To ensure successful implementation of clinical trials in low-resource settings such as SSA, studies with a simple design should be started to provide a learning curve for conducting clinical trials in the region.^2^ The ARETTA study is a good example of such a study. This study will provide for the first time, to our knowledge, efficacy data on oncology treatment of solid tumors among adults in the SSA population. Toxicity, pharmacokinetic, and QoL data will also be generated. Biosamples will be obtained for archiving for future correlative studies that will further assist in the discovery of other indices relevant to breast cancer in Nigeria. Lessons learned during the implementation of this clinical trial will be valuable toward improving clinical trial activities in SSA, and this information may also be applied to lower-resource settings in the United States.

This study will determine the efficacy and toxicity of 4 cy-cles of neoadjuvant docetaxel chemotherapy given every 3 weeks in conjunction with subcutaneous trastuzumab (Herceptin SC) followed by 1 year of subcutaneous tras-tuzumab in Nigerian women with HER2-positive breast cancer. The pharmacokinetic profiles of these drugs will also be determined. Currently, there is a scarcity of data on the response rate, toxicity, and pharmacokinetic profiles of these agents in the indigenous Black population. Of note is the fact that few people can afford HER2-targeted therapy in Nigeria or even standard chemotherapy without obtaining financial support from outside sources. The standard of chemotherapy care in Nigeria for HER2-positive breast cancer is anthracycline (doxorubicin-epirubicin with cyclophosphamide with or without fluorouracil [AC-EC-FEC]) for 4 courses, followed by a taxane with trastuzumab. This regimen is given in the adjuvant setting. In this study, neoadjuvant treatment will be given to assess chemosensitivity of the systemic agents, namely, docetaxel and trastuzumab. FEC will be introduced if there is an unsatisfactory response to docetaxel and trastuzumab. Because such patients would already be receiving trastuzumab, it would to be continued. A previous protocol using neoadjuvant paclitaxel given once per week plus trastuzumab followed by concurrent epirubicin (75 mg/m^2^ once every 3 weeks plus trastuzumab) resulted in no patient developing drug-induced congestive cardiac failure.^6^ However, to minimize toxicity, only 3 courses of FEC instead of 4 will be given in this study to drastically reduce the cumulative dose of epirubicin, which is less cardiotoxic than doxorubicin.

Few patients in Nigeria have been able to afford a complete course of trastuzumab (18 cycles) so far. Most patients take a few courses and default because of lack of money.^7^ Patients with cancer in Nigeria pay for cancer treatment out of pocket. In most instances, because of the high cost of cancer treatment, most patients cannot afford treatment and therefore seek financial support from family members and philanthropists. If these are not forthcoming, they abandon the treatment. Health insurance in Nigeria, which covers approximately 5% of the population, provides limited support to patients with cancer.^8^ This often leads to suboptimal therapy. Under such conditions, retrospective data on the response rate and toxicity of chemotherapy agents are not reliable. In this study, optimal therapy will be provided. This will lead to the acquisition of complete data for analysis. The aim of this protocol is to study the effect of docetaxel and trastuzumab combination (response rate and toxicity) on the indigenous African population. The resulting data will be used to support advocacy activities to draw support from public and private sectors toward supporting effective treatment of patients with HER2-positive breast cancer in Nigeria.

## ARETTA STUDY DESIGN

ARETTA (ClinicalTrials.gov identifier: NCT03879577) is a 1-stage, phase II, multicenter feasibility and suitability study to evaluate the efficacy and toxicity of docetaxel given every 3 weeks for 4 cycles plus trastuzumab in 60 previously untreated women with nonmetastatic breast cancer. The primary endpoint is to assess the proportion of patients with complete pathologic response (pCR) at surgery. Secondary endpoints include the number of patients who required dose delays in docetaxel and trastuzumab attributed to hematologic, GI, and cardiac toxicity. Pharmacokinetic profiles of trastuzumab will also be described. The study has been approved by the institutional review boards of the 4 participating centers, as well as the National Agency for Food and Drug Administration, the body responsible for food and drug administration in Nigeria.

The study considers docetaxel given every 3 weeks for 4 cycles as recommended backbone to be combined with subcutaneous trastuzumab.^9^ This combination has been shown to be efficacious with manageable toxicities in other populations. One of the objectives for this biomarker-stratified phase II feasibility/suitability study is to establish a platform for clinical trials in Nigeria and identify genomic biomarkers of response and toxicity to protocol-driven and optimal HER2-targeted therapy in Nigerian women. The biomarker of interest in this study is HER2 protein. The expression of HER2 protein on breast cancer samples will be used to select patients with breast cancer for participation in this study because trastuzumab, an investigational medical product, is a targeted therapy against HER2-positive breast cancer. Taxane given once every 3 weeks has been reported to be well tolerated by elderly patients with breast cancer, and one of our objectives is to use this clinical trial to improve management of chemotherapy toxicities in patients undergoing treatment of cancer.^10^

Breast ultrasound will be performed at baseline and after every 2 cycles (every 6 weeks) during neoadjuvant chemotherapy. Patients with complete clinical response (CCR) after breast ultrasound assessment after 4 cycles of docetaxel plus subcutaneous trastuzumab will undergo surgery. At surgery, those with pCR will not have additional chemotherapy, and they will receive 1 year of subcutaneous trastuzumab to complete 18 cycles. Those with partial response (PR) or stable disease (SD; operable) based on RECIST criteria version 1.1 (using breast ultrasound measurements) will be switched to have FEC plus subcutaneous trastuzumab for an additional 3 cycles in the neoadjuvant setting. Breast ultrasound will be repeated, and those with clinical response (CCR- or PR-operable) will undergo surgery and continue to complete 18 cycles of subcutaneous trastuzumab after surgery within 1 year. Patients with SD (inoperable) after salvage FEC plus subcutaneous trastuzumab or patients with progressive disease (PD) before salvage FEC plus subcutaneous trastuzumab will go off study treatment and have additional therapy by the managing physician and will be followed for the survival endpoint. Adjuvant treatment will include hormonal therapy for hormone receptor–positive disease. Radiation therapy will be administered per standard of care.

The primary endpoint is pCR in the 1-stage phase II design. All patients will have active follow-up every 3 months and have routine blood tests and physical examination for the first 2 years and every 6 months for 10 years. Computed tomography scans and bone scans will be performed as clinically indicated during the follow-up period to evaluate recurrence.

### Treatment

**FIG 1:**
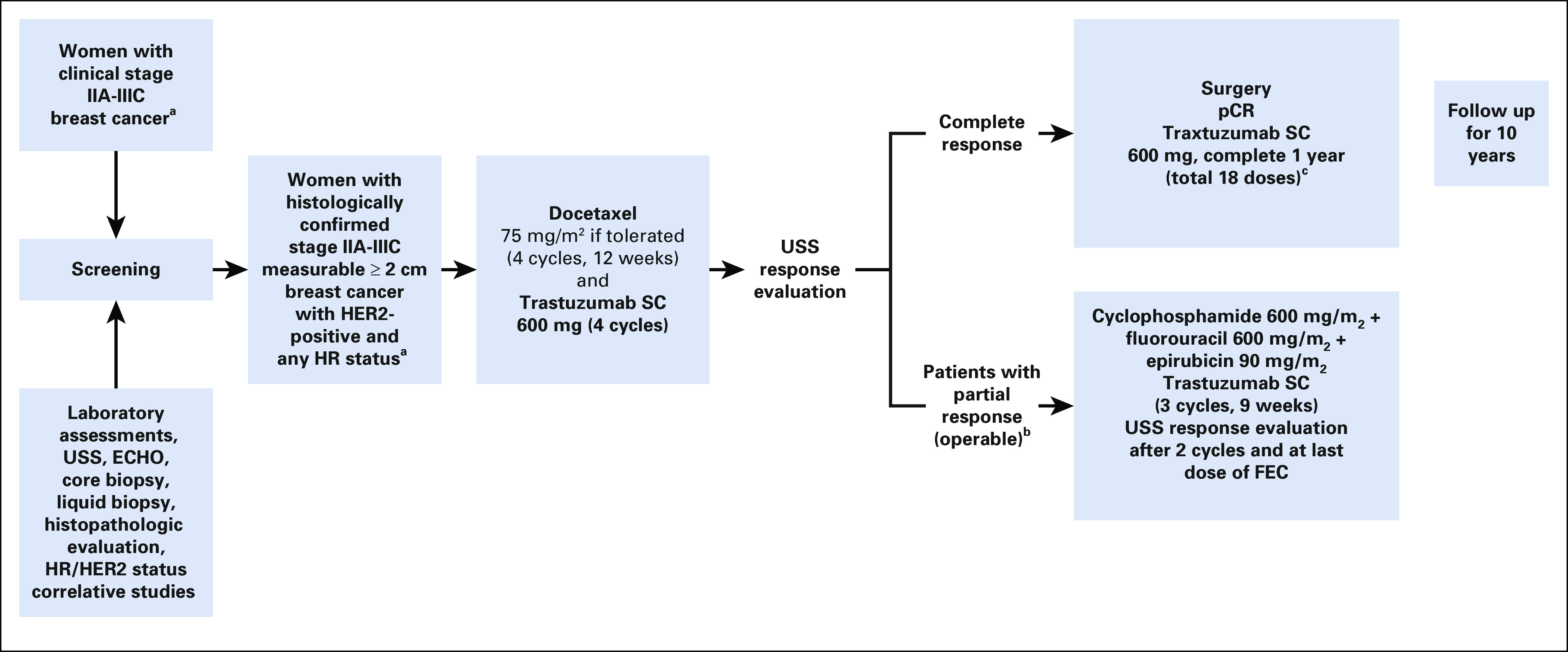
Schema showing the treatment schedule for patients in the ARETTA study. Docetaxel (T): given once every 3 weeks for a total of 4 cycles to be administered with trastuzumab. FEC: given once every 3 weeks for a total of 3 cycles to be administered for partial responders before surgery. Trastuzumab (Herceptin): given once every 3 weeks for a total of 4 cycles with docetaxel (or 7 doses for patients receiving FEC) preoperatively. Hormone receptor–positive patients will receive hormonal therapy. After surgery, all patients will continue trastuzumab to complete 1 year. (^a^) Women must also meet other inclusion criteria (listed in Key Inclusion Criteria). (^b^) Inoperable patients and patients with progressive disease will be taken off the study. (^c^) Patients with residual disease at surgery may receive additional chemotherapy at the discretion of the treating oncologists if hormone receptor negative. Hormone receptor positive patients will receive hormonal therapy. After surgery, all patients will continue trastuzumab to complete 1 year. ECHO, echocardiography; FEC, cyclophosphamide, fluorouracil, and epirubicin; HER2, human epidermal growth factor receptor 2; HR, hormone receptor; LHRH, luteinizing hormone-releasing hormone; pCR, complete pathologic response; SC, subcutaneous; USS, ultrasound scanning.

All patients will commence per-protocol treatment within 2 weeks of diagnosis. We plan to treat patients with docetaxel plus subcutaneous trastuzumab given every 3 weeks for a total of 12 weeks per the schema in Figure 1. Ultrasonography for response assessment will be performed every 6 weeks, and patients achieving CCR after 4 cycles of docetaxel plus subcutaneous trastuzumab will undergo surgery. Surgery will be performed in the fourth week after the last cycle of chemotherapy. Adjuvant hormone therapy is recommended for 5-10 years for all estrogen receptor–positive (ER+) or progesterone receptor–positive (PgR+) patients after the completion of surgery and chemotherapy (ER+/PgR+ defined as ≥ 1% of stained nuclei/hpf). Hormone receptor status will be determined at initial core biopsy for assignment of targeted therapy with tamoxifen or aromatase inhibitor of choice, and it will be reassessed in resected tumors. Tamoxifen (20 mg orally once daily) or the aromatase inhibitor letrozole (2.5 mg orally once daily; for documented postmenopausal women only)^11^ will be started within 12 weeks of completion of chemotherapy. This allows for the variable period needed for surgery to be completed. All premenopausal patients will receive the luteinizing hormone-releasing hormone (LHRH) agonist goserelin (Zoladex) 10.8 mg (subcutaneously) every 3 months for 2 years from the onset of study treatment. Addition of LHRH agonists to tamoxifen, chemotherapy, or both, has been reported to reduce disease recurrence and death after recurrence in premenopausal women with hormone receptor–positive breast cancer.^12^ Furthermore, when fertility issues are a concern, the use of LHRH agonists has been recommended in young women with breast cancer when other more effective fertility preservation methods, such as unfertilized ova cryopreservation with in vitro fertilization, embryo cryopreservation, and ovarian tissue cryopreservation, are not feasible.^13^ In our setting, these methods are not available. QoL assessment using the European Organisation for Research and Treatment of Cancer QoL instrument (English or Yoruba language versions) will be administered for all participants before each chemotherapy session.

In this study, we consider docetaxel given every 3 weeks as recommended backbone to be combined with subcutaneous trastuzumab, with a manageable toxicity rate. This combination has been shown to be efficacious with manageable toxicities in other populations and is also tolerated by elderly patients. Docetaxel is a highly effective agent in metastatic breast cancer. Response rates and duration of response and survival are improved when trastuzumab is combined with chemotherapy. Furthermore, preclinical reports demonstrate synergistic cytotoxic activity when docetaxel and trastuzumab are combined. Their different mechanisms of action and a nonoverlapping toxicity profile make this combination attractive for minimizing potential cardiotoxicity.^14^ Although many guidelines recommend the use of doxorubicin and cyclophosphamide for 4 cycles followed by docetaxel with subcutaneous trastuzumab, we chose to start with docetaxel and subcutaneous trastuzumab in the neoadjuvant setting. If there is a pCR, the patients will not need to continue with additional toxic regimen, which is one of the factors that most Nigerian women are afraid of while receiving chemotherapy. Docetaxel will be given at 75 mg/m^2^, increasing to 100 mg/m^2^ from cycle 2 onward if the 75-mg starting dose was well tolerated (by intravenous infusion and diluted in normal saline [NS] or 5% dextrose in water [D5W]) and given every 3 weeks for 4 cycles.^15^ Patients with CCR will have surgery within 4 weeks of the last dose of docetaxel plus trastuzumab. Patients with PR or SD will continue with 3 doses of FEC (3 cycles of intravenous FEC [fluorouracil 600 mg/m^2^, epirubicin 90 mg/m^2^, and cyclophosphamide 600 mg/m^2^, diluted in NS or D5W]) plus trastuzumab every 3 weeks before surgery. Six courses of epirubicin have been combined with trastuzumab in metastatic breast cancer with a lower dose-limiting cardiotoxicity of 1.7% compared with the doxorubicin arm with a dose-limiting toxicity of 5%.^16^ In this study, we use a lesser dose (3 courses) to improve cardiotoxicity and to enlist the good response of breast cancer to an anthracycline-based regimen after poor response to first-line docetaxel.^6^ While receiving chemotherapy, patients with an absolute neutrophil count (ANC) between 1,000 and 1,500 will receive filgrastim with chemotherapy, whereas those with ANC < 1,000 will receive filgrastim and wait until ANC is ≥ 1,000 before additional chemotherapy.

All patients will receive anti-HER2 blockade trastuzumab. Trastuzumab therapy will be given at 600 mg subcutaneously (fixed dose irrespective of patients’ weight/body surface area) on day 1 of each cycle and thereafter every 3 weeks. A total of 18 doses will be given (1 year). Trastuzumab dosing is not interrupted during surgery. The doses and treatments, as well as dose adjustment schedules, are presented in Tables 1 and 2.

**TABLE 1:**
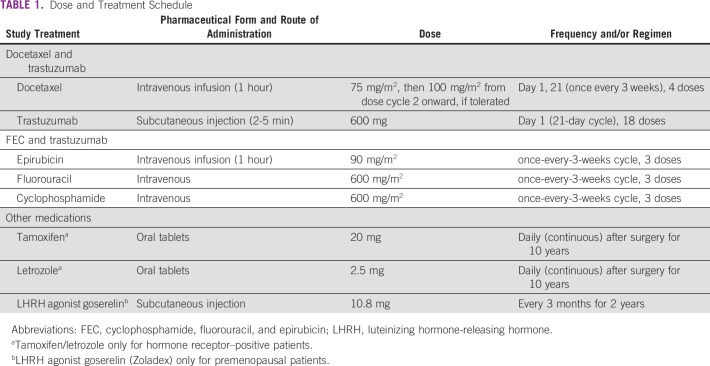
Dose and Treatment Schedule

**TABLE 2:**
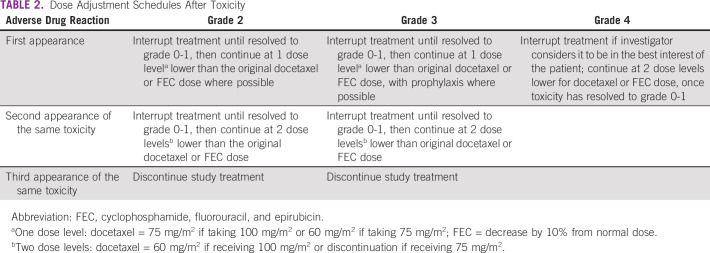
Dose Adjustment Schedules After Toxicity

### Toxicity Assessment

#### Toxicity will be assessed using National Cancer Institute–Common Terminology Criteria for Adverse Events (version 5).

Toxicity due to docetaxel or FEC may be managed by symptomatic treatment, dose interruptions, and adjustment of docetaxel-FEC dose. Weekly reassessment of toxicities will be performed to guide appropriate dose adjustments. The schedule for dose adjustments for the chemotherapy regimens is provided in Table 2. Doses of medications omitted for toxicity are not replaced or restored; instead, the patient should resume the planned treatment cycles. The omitted doses will be noted as treatment interruptions. Patients who miss their treatment doses for more than 2 consecutive weeks will be classified under treatment interruptions and will not be analyzed as per-protocol treatment. There is no dose modification for subcutaneous trastuzumab

#### Cardiomyopathy.

We will assess for symptoms before each cycle and evaluate cardiac toxicity by echocardiography every 12 weeks during neoadjuvant treatment and every 12 weeks during adjuvant trastuzumab. Treatment with trastuzumab will be stopped for a minimum of 3 weeks (1 cycle) for any of the following: if left ventricular ejection fraction (LVEF) is < 50% with ≥ 15% decrease from baseline; if LVEF returns to 50% or higher, treatment can be resumed; if a decrease in LVEF persists or has declined further, or if clinically significant congestive heart failure has developed, treatment with trastuzumab will be stopped; and if, after a repeat assessment within approximately 3-7 weeks (2 cycles), the LVEF has not improved or has declined further, trastuzumab will be discontinued.

#### Correlative studies.

Tissue samples collected under this study will also be used by the University of Chicago with Nigerian investigators to generate additional biomarkers and molecular data on next-generation technology platforms and future studies on breast cancer in Nigerian women. These molecular characteristics (biomarkers/pathways) will be used for better stratifying tumor type and to correlate with pathologic, imaging, or response to treatment measures. During the trial, standard and exploratory biomarkers will be tested for their ability to predict tumor response to specific classes of investigational targeted therapeutics.

### Key Inclusion Criteria

Women ages 18 to 70 years who have biopsy-accessible breast tumor of significant size for core needle biopsy/ultrasound measurable (≥ 2 cm) and with histologically confirmed carcinoma of the female breast with 3+ positive HER2 status by immune histochemistry (IHC) are included. At present, there are no facilities for fluorescence in situ hybridization/in situ hybridization testing in Nigeria for the confirmation of equivocal HER2 status (2+ by IHC). Such samples are usually sent outside the country for confirmation if the individual can afford to pay for the services. Otherwise, additional treatment is usually at the discretion of the treating physician. However, if a participant with equivocal IHC result can afford FISH/ISH testing and the result is positive for HER2 overexpression, the patient will be eligible to participate in the study.

Participants should have clinical stages IIA-IIIC (American Joint Committee on Cancer 2009) and be chemotherapy naïve. They also should have Eastern Cooperative Group performance status 0-2; echocardiogram baseline LVEF ≥ 55%; adequate bone marrow function defined as ANC ≥ 1,000/mm^3^; platelets ≥ 100,000/mm^3^; and hemoglobin ≥ 10 g/dL or packed cell volume 30%.

### Key Exclusion Criteria

Pregnant or lactating women and those with distant metastasis (brain and/or visceral metastasis), with HER2-negative disease, or receiving treatment for other carcinomas within the last 5 years are excluded. Other exclusion factors include presence of other cardiac conditions that may increase the risk of cardiac compromise with trastuzumab. These include poorly controlled blood pressure (> 140/85 mmHg), significant valvular heart disease (presence of murmurs on auscultation), signs of heart failure (raised venous pressure, crepitations over the lung fields, or pedal edema), gross abnormalities in a 12-lead electrocardiogram such as arrhythmias (atrial fibrillation, atrial flutter, heart block), and evidence of previous myocardial infarction (Q waves, left bundle branch block).^17^

### Statistical Considerations

All patients who signed consent and initiated neoadjuvant therapy will be included in the analysis. Demographics and baseline features will be presented using descriptive statistics. Based on a current review of the literature in other populations, for this study, we expect an inefficient treatment regimen of docetaxel plus subcutaneous trastuzumab to elicit 20% or fewer pCRs (p0 = 0.20) and a successful treatment regimen to have at least 40% pCR at surgery (p1 = 0.40; p0 being the response proportion {pCR rate} that if true,clearly implies that the treatment does not warrant further study and p1 being the response proportion that if true would imply that the treatment has sufficient efficacy to warrant further trials). The patients with SD or PR who attain pCR after an additional 3 courses of FEC will be counted as successes, whereas those who are declared inoperable or have PD will be considered failures. A 1-sided binomial test will be performed to test the null hypothesis of a 20% pCR rate versus a 40% alternative.

Secondary analyses will compare pCR rates by hormone receptor status, lymph node status, and breast cancer subtype using Fisher’s exact test. Disease-free survival (DFS) will be defined as the time from enrollment to first occurrence of any of the following events: recurrence of ipsilateral invasive breast tumor, recurrence of ipsilateral locoregional invasive disease, distant recurrence, contralateral invasive breast cancer, or death from any cause. DFS will be estimated by the Kaplan-Meier method; prognostic factors will be identified using Cox regression. The frequency of toxicity by type and grade will be summarized. Mixed effects regression models will be fit to analyze QoL.

## PERSPECTIVES OF CANCER CLINICAL TRIALS IN SSA

The ARETTA protocol conforms with most of the considerations suggested on how to design trials toward increasing cancer clinical trials in SSA. A report by Gupta et al^18^ identified opportunities for clinical trials on cancer in developing countries, such as those in SSA. There are large numbers of patients who receive less intense treatments, thereby providing opportunities to try other agents, and trials using the neoadjuvant approach are particularly suitable. The ARETTA protocol will recruit treatment-naïve participants with nonmetastatic disease in the neoadjuvant setting. They also reported that clinical and translational research collaboration between centers in developed and developing countries is important for building capacity. The ARETTA protocol was developed by investigators from Nigeria but with guidance and input from collaborators from Novartis Institute for Biomedical Research, University of Chicago, and Roche, which are institutions from high-income countries. Specimens collected from the trial will be analyzed for collaborative studies at the University of Chicago. Gopal^19^ proposed key attributes of cancer clinical trials in SSA, as shown in Table 3.

**TABLE 3:**
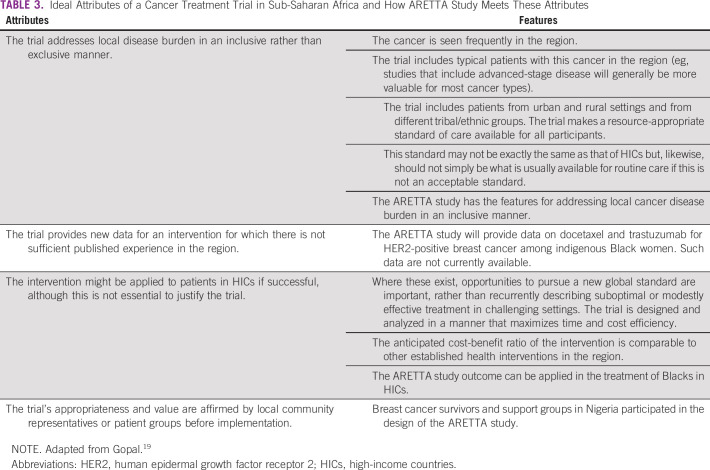
Ideal Attributes of a Cancer Treatment Trial in Sub-Saharan Africa and How ARETTA Study Meets These Attributes

### Protocol Suitability

The concept of protocol suitability is different from feasibility. In contrast to “feasible,” which is defined as achievable and possible, “suitable” means appropriate. Protocol suitability surpasses feasibility by addressing not only technical aspects of the protocol but also considers study settings, environments, and culture, as well as effectiveness and efficiency of execution.^20^ Protocol suitability can therefore be regarded as fit for purpose. This ensures minimal protocol deviation, which confers study integrity. Involvement of trial staff in protocol development has been stated as a key factor in ensuring protocol suitability. The ARETTA protocol development involved team members from the 4 participating institutions, thereby ensuring feasibility and suitability.

Indigenous Black women of SSA are under-represented in clinical trials. None of the current drugs used for breast cancer in the subregion were tested among SSA women during the drug development processes. The design of a trial such as the ARETTA study, which takes into consideration the peculiarities of patient characteristics and available resources and infrastructure, will hopefully address barriers to participation of SSA women in global clinical trials and answer the question of the hematologic, neurologic, and cardiac safety of docetaxel and trastuzumab in indigenous Blacks from SSA. QoL issues as well as pharmacokinetic profiles of trastuzumab will also be described. The results of this novel trial will provide an important contribution toward narrowing the gap in cancer clinical trial disparities and further advance the goal of global personalized medicine.
